# Optimization of Mix Proportions for Type IV Cement-Based Grouting Materials Using Orthogonal Experimental Design

**DOI:** 10.3390/ma19153328

**Published:** 2026-08-05

**Authors:** Binghao Wang, Yong Xu, Yingda Zhang, Ziqiang Lyu, Zhen Wang, Meiling Gu

**Affiliations:** 1 Chengdu Urban Construction Investment Management Group Co., Ltd., Chengdu 610037, China; 2 School of Architecture and Civil Engineering, Xihua University, Chengdu 610039, China; 3 Chengdu Longfor Property Services Co., Ltd., Chengdu 610011, China

**Keywords:** type IV cement-based grout, orthogonal experiment, mix proportion, compressive strength, fluidity

## Abstract

This study systematically investigates the effects of water-to-binder ratio, total binder content, silica fume content, and fly ash content on the fluidity and compressive strength of Type IV cement-based grouting materials. An L_16_(4^4^) orthogonal experimental design was adopted to evaluate the influence of these four factors on initial and 30 min slump flow, as well as 1-day, 3-day, and 28-day compressive strength. The range analysis results indicate that silica fume content exerts the most significant effect on initial slump flow, while the water-to-binder ratio predominantly governs the 30 min slump flow and early-age compressive strength, such as at 1 day and 3 days. The total binder content is identified as the key factor influencing the 28-day compressive strength. Furthermore, supplementary tests on the combined incorporation of fly ash and silica fume reveal that silica fume enhances early-age strength but reduces fluidity, whereas fly ash improves fluidity but promotes later-age strength development, with an optimal substitution range for both admixtures. Microstructural analyses including XRD and SEM and hydration heat tests further elucidate the underlying mechanisms, demonstrating that the effects of the two admixtures optimize the hydration process and microstructure densification. Based on the comprehensive evaluation, the optimal mix proportion was determined as A_3_B_2_C_4_D_2_, corresponding to a water-to-binder ratio of 0.31, a fly ash content of 15%, a total binder content of 690 kg/m^3^, and a silica fume content of 8.5%. This optimized formulation achieves a balanced performance in both workability and strength development.

## 1. Introduction

Cement-based grouting materials are critical construction materials widely used in structural reinforcement, foundation repair, and prestressed duct filling [1,2]. Their performance directly impacts the durability and safety of structures [3,4]. Grouting materials are categorized into Types I, II, III, and IV, with Type IV grouts being subject to strict standards regarding aggregate particle size with a nominal lower limit above 4.75 mm and upper limit below 25 mm [5]. Consequently, they are extensively applied in the strengthening and modification of concrete and masonry structures. As engineering demands evolve towards high fluidity, high strength, and long service life, the development of Type IV cement-based grouts characterized by high strength and ultra-high performance has garnered significant attention [6,7].

Compared with fine cementitious grouts, Type IV cement-based grout contains coarse aggregate and is commonly used in structural strengthening, equipment-foundation rehabilitation, concrete repair, and other applications involving relatively large filling spaces. The presence of coarse aggregate improves dimensional stability and reduces material cost, but also introduces additional challenges related to aggregate suspension, segregation resistance, flow retention, and interfacial bonding. Consequently, the mixture design of Type IV grout must simultaneously satisfy high filling capacity, adequate workability retention, rapid early-age strength development, and sufficient later-age mechanical performance. These requirements make the optimization of Type IV grout more complex than that of conventional fine-particle grouting materials.

The performance of these grouts is influenced by the effects of multiple factors, including the water–binder ratio (W/B), binder composition, and mineral admixtures such as silica fume (SF) and fly ash (FA) [8,9]. However, existing research still lacks comprehensive optimization considering the effects among these multiple factors, particularly in the systematic analysis of fluidity and strength development at different ages.

Recent studies indicate that the W/B ratio is a core parameter affecting the fluidity and strength of grouts. Zhu et al. [10] found through orthogonal experiments that the W/B ratio significantly influences density, fluidity, bleeding rate, and uniaxial compressive strength. They also noted that fly ash helps improve paste stability and workability and reduces bleeding rate without compromising fluidity. Mazloom et al. [11] indicated that the high reactivity of silica fume can significantly enhance the 28-day compressive strength and modulus of rupture, but excessive incorporation may reduce workability. Haustein et al. [12] demonstrated the feasibility of using fly ash microspheres (FAMs) as mineral admixtures in concrete, showing that their particle size significantly affects pozzolanic activity, pore structure, and mechanical properties, with small-sized FAMs (less than 200 µm) exhibiting superior performance in long-term strength development.

Previous studies have utilized orthogonal experimental design to analyze the role of certain factors [13,14]. However, systematic investigations specifically addressing Type IV grout remain limited. In particular, the relative main effects of water-to-binder ratio, total binder content, fly ash content, and silica fume content on both flow retention and strength development at different ages have not been sufficiently clarified. Moreover, few studies have combined multi-factor mixture screening with verification tests, hydration calorimetry, XRD, and SEM analyses to interpret the fresh, mechanical, and hydration behavior of coarse-aggregate cementitious grout. This is particularly true regarding the evolution of early strength, including at 1-day and 3-day ages, and at long-term ages such as 28 days, where clear conclusions are yet to be established.

This study employs the orthogonal experimental method to systematically investigate the effects of four factors including water–binder ratio, total binder content, silica fume content, and fly ash content on the initial and 30 min slump flow, as well as the 1-day, 3-day, and 28-day compressive strength of Type IV cement-based grouting materials. Additional fly ash–silica fume proportioning tests were performed to examine the performance of selected mineral-admixture combinations under a fixed water-to-binder ratio and binder content. Isothermal calorimetry was used to characterize early hydration kinetics, while XRD and SEM analyses were employed to examine hydration-product evolution and microstructural development. The expected outcome was to identify a practically balanced mixture and clarify the main material-related mechanisms governing its workability and strength development.

## 2. Experimental Program

### 2.1. Materials

**Figure 1:**
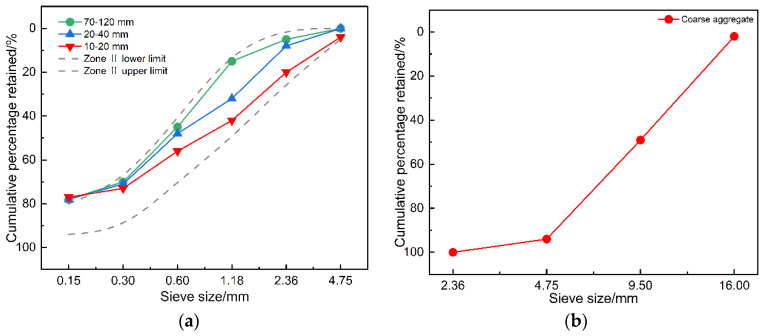
Particle size distribution of coarse and fine aggregate.

The raw materials utilized in this experimental study comprised Ordinary Portland Cement (P.O 42.5R) as the primary cementitious material, the physical and mechanical properties of which are listed in Table 1. The water demand for standard consistency of the P.O 42.5R cement was 26.9%, according to the manufacturer’s quality inspection certificate. The compositional indices of the cement are shown in Table 2. Class I FA obtained from a power plant in Jiangyou (Mianyang Zhennan Environmental Protection Building Materials Co., Ltd., Mianyang, China) and SF90-grade SF sourced from a Chengdu supplier (Chengdu Yanzhu Jiuzhu Building Technology Co., Ltd., Chengdu, China) were incorporated as mineral admixtures, with their respective properties detailed in Table 3 and Table 4. The materials in this study are identical to those in the previous study of the authors [5]. The aggregates included manufactured sands with varying fineness moduli including coarse sand at 2.6, medium sand at 2.4, and fine sand at 2.1, along with 5–10 mm pea-shaped crushed stone as the coarse aggregate. The particle size distributions of coarse and fine aggregates are presented in Figure 1. Three chemical admixtures were also used in all mixtures. A GK-SP expansive agent supplied by a building-material technology company in Beijing, China, had a fineness of 200 m^2^/kg and appeared as a bright-yellow powder. It was incorporated at a fixed dosage of 0.03% by mass of the total binder. A DF04 powdered defoamer supplied by Shanghai Yingshan New Material Technology Co., Ltd., (Shanghai, China), appeared as a light-gray to white powder and had a moisture content of 2.3%, a pH of 8.6, and a bulk density of 375.1 kg/m^3^. Its dosage was fixed at 0.11% by mass of the total binder. A polycarboxylate-based superplasticizer supplied by Sichuan Lujia Sitong Technology Development Co., Ltd., (Chengdu, China), was incorporated at a fixed dosage of 0.32% by mass of the total binder. The dosages of the expansive agent, defoamer, and superplasticizer were kept constant in all orthogonal-design mixtures and subsequent mineral-admixture proportioning tests.

### 2.2. Test Method and Experimental Design

The fluidity of the grouting materials was evaluated by the slump flow test, conducted in accordance with the Chinese national standard “Technical code for application of cementitious grout” (GB/T 50448-2015) [15]. These tests were used as empirical engineering indicators of flowability and flow retention. It should be noted that slump-flow diameter does not provide direct measurements of yield stress, plastic viscosity, thixotropy, structural build-up, or particle-dispersion state. Before mixing, the mixing bowl and blades were moistened with a damp cloth, and any visible free water was removed. The pre-weighed coarse aggregate, fine aggregate, and binder materials were sequentially introduced into an SJD-60 compulsory mixer. The mixer had a rated power of 1.5 kW and a rotational speed of 48 r/min. The dry materials were initially mixed for 3 min, after which the mixing water and pre-weighed chemical admixtures were added, followed by an additional 5 min of mixing. The compressive strength was determined on 150 mm cubic specimens following the procedures specified in the “Standard for test methods of concrete physical and mechanical properties” (GB/T 50081-2019) [16].

An orthogonal experimental design, a mathematical method for efficiently planning multi-factor experiments, was employed to identify the primary and secondary influencing factors and to determine the optimal mix proportion [17]. In this study, four factors were investigated: water-to-binder ratio (Factor A), fly ash content (Factor B), total binder content (Factor C), and silica fume content (Factor D). Each factor was set at four levels, as presented in Table 5. The L_16_(4^4^) orthogonal array was selected as a first-stage screening design because four factors were investigated at four levels. A full factorial design would require 4^4^ = 256 mixture combinations, whereas the adopted array reduced the number to 16 while maintaining balanced level allocation. The design was intended primarily to estimate and compare the main effects of the four factors. The water-to-binder ratio was deliberately treated as an experimental factor rather than a fixed parameter. Four levels of 0.27, 0.29, 0.31, and 0.33 were selected based on preliminary trial mixtures. For each mix, three 150 mm cubic specimens were cast per test age, leading to a total of 144 specimens for compressive strength measurement at 1, 3, and 28 days. All mixes were prepared using a compulsory mixer. After casting, the specimens remained in the molds for 24 h under laboratory conditions of 20 ± 5 °C and 65 ± 5% relative humidity. The exposed surfaces were immediately covered with plastic film to minimize moisture exchange with the surrounding environment. After 24 h, the specimens were demolded and transferred to a standard curing chamber maintained at 20 ± 2 °C and a relative humidity above 95% until the designated testing ages.

### 2.3. Microstructural Analysis

To elucidate the underlying mechanisms governing macroscopic performance including fluidity and mechanical strength of the optimized grouting materials, microstructural analyses were conducted on selected representative samples. The mineralogical compositions of the cementitious paste were identified using X-ray diffraction (XRD) analysis over a 2θ range of 5° to 70° with a scanning step of 0.2°. At the designated age, representative paste fragments were immersed in absolute ethanol for 12 h to stop hydration and then dried at 60 °C. The dried samples were ground using a ball mill and passed through a 1 µm sieve. The powders were stored in sealed containers before testing to minimize moisture exposure and carbonation. The morphology and microstructure of the hydration products were observed via scanning electron microscopy (SEM) at an acceleration voltage of 5 kV. Prior to observation, the specimens were vacuum-dried and gold-coated to enhance conductivity. XRD and SEM analyses were conducted on selected samples cured for 1, 3, and 28 days to examine the evolution of crystalline phases and microstructural features with curing age to correlate the strength development and fluidity behavior with the microstructural characteristics, such as the formation of calcium silicate hydrate (C-S-H) gel, the consumption of portlandite (Ca(OH)_2_), and the overall matrix densification.

### 2.4. Hydration Heat Test

Isothermal calorimetry was conducted using a TAM air calorimeter at 20 ± 1 °C. For each test, 10 g of total binder and the corresponding amount of water were mixed using a manual mixing method for 60 s. The freshly mixed paste was immediately transferred into a 15 mL glass ampoule, sealed with a top, and placed in the calorimeter within approximately 5 min after initial water–binder contact. The isothermal calorimeter runs at a constant temperature of 20 ± 1 °C. The heat flow rate and cumulative hydration heat were continuously recorded for 72 h immediately after the mixing of each sample with water. The water-to-binder ratio and total binder content were kept consistent with those of the corresponding mix proportions.

## 3. Results and Discussions

### 3.1. Analysis of Slump Flow Results

The performance of the prepared grouting mixtures was assessed against the specifications for Type IV cement-based grouts outlined in the Chinese standard “Cementitious grout” JC/T 986-2018 [18,19]. This standard mandates that the minimum values for the initial slump flow are higher than 650 mm and those for the 30 min slump flow are higher than 550 mm. The compressive strength requirements must conform to the criteria presented in Table 6 as per DBJ53/T-173-2025 [20]. The mix design and the detailed orthogonal experimental design (L_16_(4^4^)) along with the corresponding test results for all 16 mixes are provided in Table 7 and Table 8. The range analysis results for the slump flow and compressive strength are summarized in Table 9 and Table 10, respectively.

The range analysis results presented in Table 9 reveal the order of significance of the factors affecting the initial slump flow: silica fume content (D) > water-to-binder ratio (A) > total binder content (C) > fly ash content (B). This indicates that the silica fume content was the most influential factor, followed by the water-to-binder ratio and total binder content as significant factors, whereas the fly ash content had a relatively minor effect. For the 30 min slump flow, the order of significance shifted to water-to-binder ratio (A) > silica fume content (D) > total binder content (C) > fly ash content (B). The water-to-binder ratio emerged as the primary factor, with silica fume and total binder content remaining important, and fly ash content still exhibiting the least influence.

**Figure 2:**
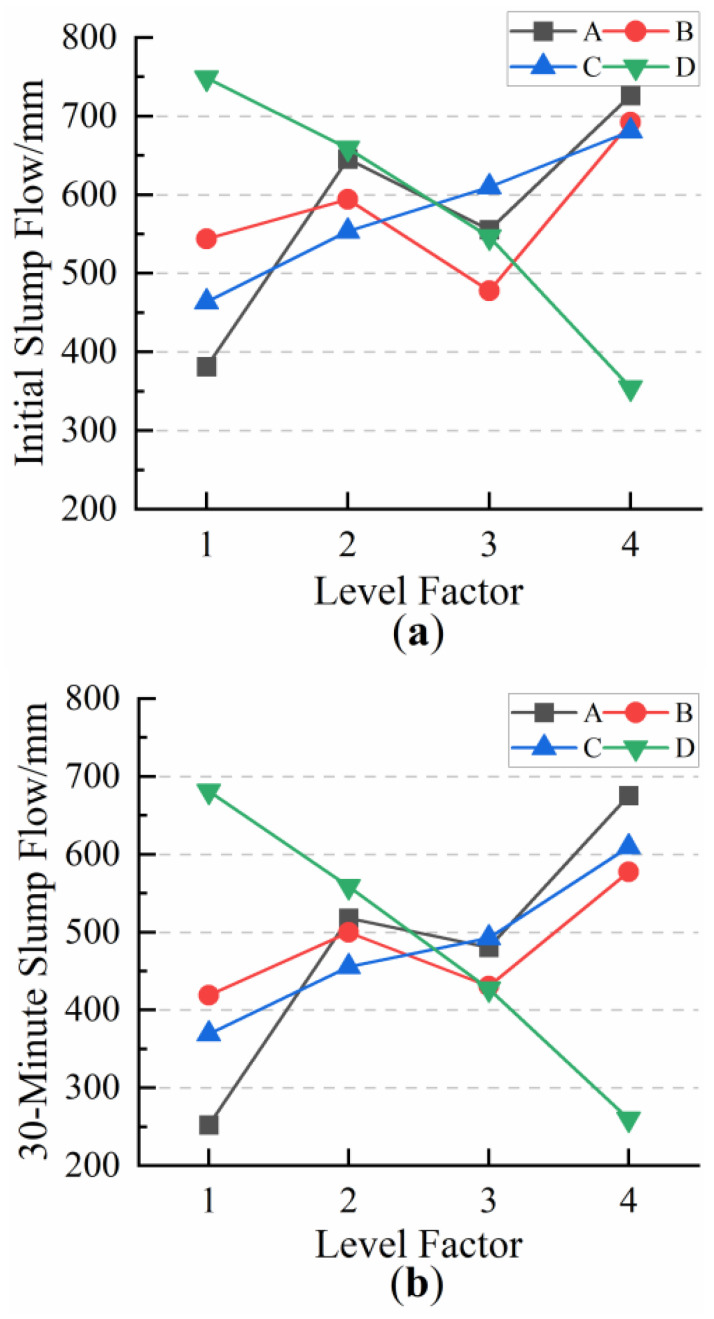
Level-average effects of the investigated factors on (**a**) initial slump flow and (**b**) 30 min slump flow.

As illustrated in Figure 2, the trends of how factors A, B, C, and D affect fluidity differ. Regarding the initial slump flow, an increase in the silica fume content (D) from 2.5% to 20.5% resulted in a 53% decrease. This is attributed to the broken chemical bonds on the surface of silica fume particles, where the fractured ends readily form Si-OH groups in water [21]. Increasing the silica fume content was associated with a reduction in the measured slump flow. This response may be related to its high fineness, increased water demand, possible particle agglomeration, and greater superplasticizer demand [22]. The initial slump flow exhibited a non-monotonic variation with the water-to-binder ratio, increasing from level 1 to level 2, decreasing at level 3, and increasing again at level 4. This trend should not be interpreted as the isolated effect of the water-to-binder ratio. In the L_16_(4^4^) orthogonal design, the average response corresponding to each level of Factor A was obtained from four mixtures with different fly ash contents, silica fume contents, and total binder contents. Therefore, the level-average curve reflects the combined response of the orthogonal factor combinations rather than a conventional single-factor relationship in which all other variables are held constant. It showed a general increasing trend with Factor C (total binder content). For Factor B (fly ash content), the initial slump flow first increased, then decreased, and finally increased once more.

For the 30 min slump flow, Figure 2b shows that it generally increased with the water-to-binder ratio (Factor A). In contrast, it decreased with an increase in silica fume content (Factor D). Similar to the initial flow, the 30 min slump flow increased with the total binder content (Factor C) and exhibited a pattern of initial increase, followed by a decrease, and a final increase with the fly ash content (Factor B). This indicates that while the influence trends of the various factors on the 30 min slump flow were similar to those on the initial flow, the primary influencing factor changed.

An increase in the water-to-binder ratio reduces the viscosity of the paste. However, the significant density difference between the coarse aggregate and the paste led to the sinking of coarse aggregate, causing segregation [23]. After 30 min, this segregation became more pronounced [24]. When the 30 min slump flow test was subsequently conducted, this enhanced segregation effect made the water-to-binder ratio with a range R = 423.13 mm the dominant factor over silica fume with R = 421.25 mm for 30 min fluidity retention.

### 3.2. Analysis of Compressive Strength Results

As derived from Table 10, the influence of the various factors on the compressive strength exhibited significant differences depending on the curing age. For the 1-day compressive strength, the order of significance was: water-to-binder ratio (A) > fly ash content (B) > silica fume content (D) > total binder content (C). This indicates that factor C exhibited the largest observed main-effect amplitude for the 28-day compressive strength within the adopted orthogonal design; fly ash and silica fume contents were significant influencing factors, while the total binder content had a relatively minor effect. For the 3-day compressive strength, the order was A > D > B > C, with the water-to-binder ratio remaining the primary factor, followed by silica fume and fly ash contents as important factors; the influence of the total binder content was still the least pronounced. In contrast, the corrected order of influence on the 28-day compressive strength was C > D > B > A. The total binder content was therefore the most influential factor, followed by the silica fume content and fly ash content, whereas the water-to-binder ratio exhibited the smallest range within the investigated levels.

**Figure 3:**
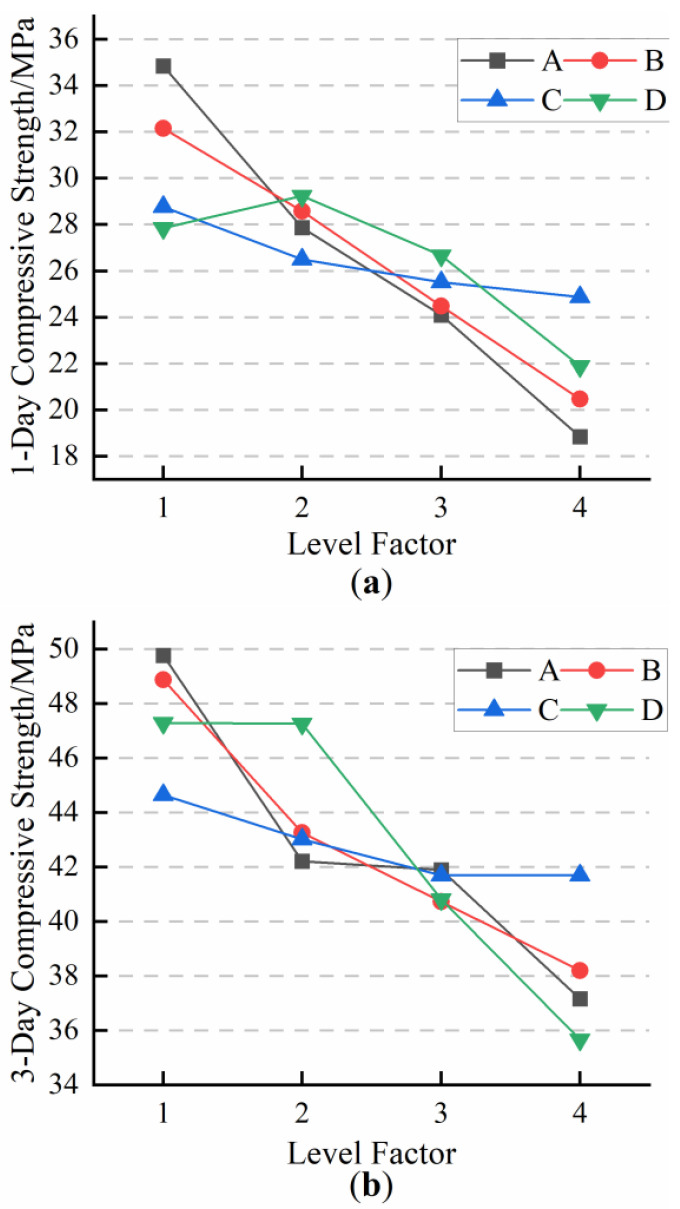

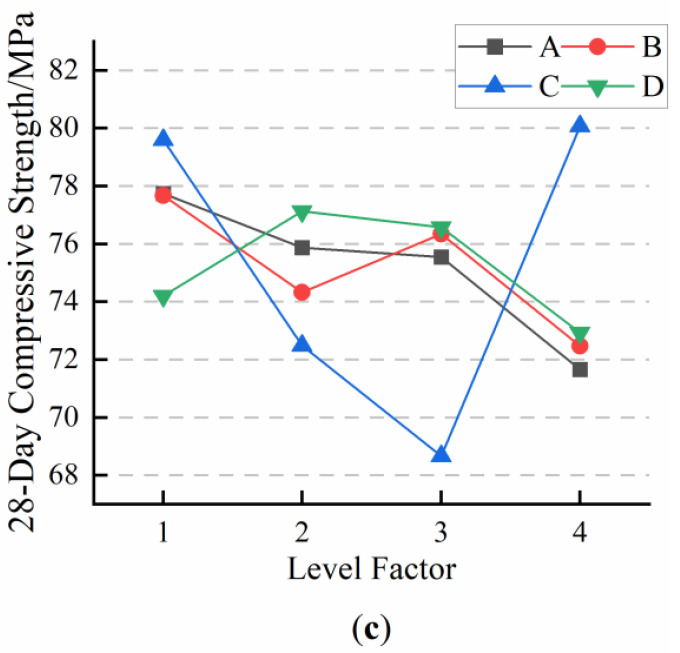
Analysis of the range of compressive strength: (**a**) 1 day; (**b**) 3 days; (**c**) 28 days.

The influence of these factors on strength development is further elucidated in Figure 3. Regarding the 1-day strength, an increase in the water-to-binder ratio (Factor A) led to a decrease in strength [25]. A lower water-to-binder ratio generally increases early-age strength by increasing the solid concentration, reducing the volume of capillary pores formed after hardening, and promoting the formation of more C-S-H gel, resulting in higher early-age strength [26]. Conversely, an excessively high W/B ratio leads to surplus water that does not participate in hydration [27]. This water evaporates during the setting stage, creating numerous pores within the cement paste matrix, which increases capillary porosity, reduces matrix density, and consequently has a detrimental effect on strength [28]. Furthermore, severe segregation caused by a high W/B ratio also contributes significantly to the reduction in 1-day strength [29]. The 1-day compressive strength decreased with an increase in fly ash content (Factor B). For silica fume content (Factor D), the 1-day strength initially increased and then decreased with its addition. Overall, the 1-day strength decreased as the levels of factors A, B, and C increased.

For the 3-day compressive strength, as observed in Figure 3b, Factor A (W/B ratio) remained the most significant, dominating the strength development at this age (range R = 12.60 MPa). However, the pozzolanic effects of silica fume (Factor D) and fly ash (Factor B) began to manifest. The 3-day compressive strength exhibited a decreasing trend with an increase in the content of Factors D, B, and C.

The influence on the 28-day compressive strength revealed a different pattern, as shown in Figure 3c. When the total binder content (Factor C) increased from 540 kg/m^3^ to 690 kg/m^3^, the 28-day strength first decreased and then increased, reaching a minimum at 640 kg/m^3^ (68.67 MPa) and a peak at 690 kg/m^3^ (80.08 MPa), with a range R = 11.41 MPa. For Factor D (silica fume content), the 28-day strength initially increased and then decreased with higher incorporation levels. As shown in Figure 3c, the 28-day compressive strength varied non-monotonically with all four factors. For Factor C, the mean strength decreased from 73.83 MPa at C_1_ to 68.65 MPa at C_3_ and then increased markedly to 78.63 MPa at C_4_, producing the largest range of 9.98 MPa. This indicates that the highest total binder content investigated was favorable for later-age strength, although the intermediate binder levels did not produce a monotonic response.

As such, the water-to-binder ratio primarily governs the 1-day and 3-day compressive strengths by controlling the early hydration reaction and porosity. In contrast, the total binder content becomes the dominant factor for 28-day strength. This shift is attributed to the long-term hydration process, the ongoing pozzolanic reactions, and the optimization of particle packing [30]. An increase in the total binder content (cement + fly ash + silica fume) provides more unhydrated particles that continue to react at later ages, generating additional hydration products, which further fill pores and enhance density [31]. As mineral admixtures, fly ash and silica fume contribute more significantly to the pozzolanic reactions at later stages, such as those at 28 days, consuming Ca(OH)_2_ and producing additional C-S-H gel, thereby improving the compactness of the matrix [32]. Furthermore, the total binder content optimizes the particle size distribution, leading to a denser packing structure and reduced porosity, which consequently enhances the long-term strength [33]. Based on the range analysis of both slump flow and compressive strength, as seen from Figure 2 and Figure 3, the optimal content for fly ash was determined to be 15%, and for silica fume, 8.5%.

### 3.3. Verification of the Recommended Factor-Level Combination

Based on the comprehensive evaluation of the initial slump flow, 30 min slump flow, and compressive strengths at 1, 3, and 28 days, A_3_B_2_C_4_D_2_ was selected as the recommended compromise factor-level combination. This combination corresponded to a water-to-binder ratio of 0.31, a fly ash content of 15%, a total binder content of 690 kg/m^3^, and a silica fume content of 8.5%.

Since A_3_B_2_C_4_D_2_ was not included in the original L_16_(4^4^) orthogonal array, an additional verification mixture, designated Z1, was prepared under the same mixing, casting, curing, and testing conditions as the orthogonal mixtures. The measured performance of Z1 including the initial and 30 min slump flows were 855 and 825 mm, respectively, while the 1-day, 3-day, and 28-day compressive strengths were 23.7, 54.8, and 80.2 MPa, respectively. These results confirm that the recommended combination achieved satisfactory flowability and strength development and met the relevant performance requirements for Type IV cementitious grout.

It should be emphasized that A_3_B_2_C_4_D_2_ was selected to balance multiple performance indicators rather than to maximize an individual response. Level C_4_ exhibited favorable performance in terms of flowability and 28-day compressive strength, while D_2_ provided an appropriate balance between early- and later-age strength. Levels A_3_ and B_2_ were selected as intermediate levels to avoid the potentially unfavorable effects associated with the extreme water-to-binder ratio and fly ash levels. Therefore, A_3_B_2_C_4_D_2_ is more appropriately described as a recommended compromise combination for multi-response performance.

For the mechanical properties, the ANOVA results presented in Table 11 were generally consistent with the factor rankings obtained from the range analysis. For the 1-day compressive strength, both methods identified the water-to-binder ratio as the dominant factor, followed by the fly ash and silica fume contents, while the total binder content showed the smallest effect. For the 28-day compressive strength, the total binder content remained the most influential factor.

A slight difference was observed for the 3-day compressive strength. The range analysis ranked the factors as A > D > B > C, whereas the ANOVA results indicated D > A > B > C based on the corresponding F-values. Nevertheless, the effects of the water-to-binder ratio and silica fume content were substantially greater than those of the fly ash content and total binder content in both analyses. This result indicates that the water-to-binder ratio and silica fume content jointly governed the development of the 3-day compressive strength. Therefore, the ANOVA results did not alter the overall identification of the governing factors obtained from the orthogonal analysis, supporting the reliability of the experimental conclusions.

As further summarized in Table 12, the residual mean squares for all performance indicators were lower than the mean squares of the principal factors, indicating that experimental variability did not determine the ranking of the factor effects. The residual sum-of-squares proportion for the 28-day compressive strength was relatively high, reaching 23.4%, suggesting that later-age strength was more susceptible to uncontrolled variations, such as differences in specimen compaction, local aggregate distribution, and long-term hydration development. Nevertheless, the statistically significant effects of the investigated factors and the overall agreement between the range analysis and ANOVA indicate that the experimental results are sufficiently reliable for the mix-proportion analysis conducted in this study.

### 3.4. Effect of Mineral Admixtures on Grout Performance

**Figure 4:**
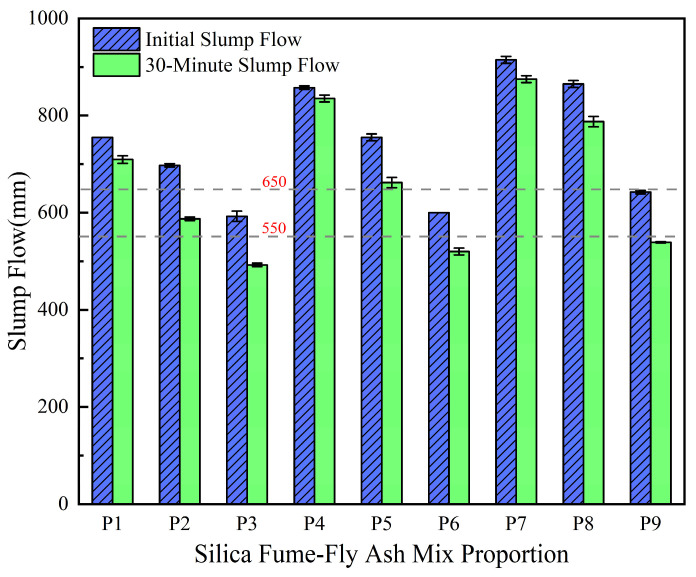
Effect of mineral admixture content on slump flow. (550 mm is the lower limit of 30-min slump flow, and 650 mm is the lower limit of initial slump flow).

**Figure 5:**
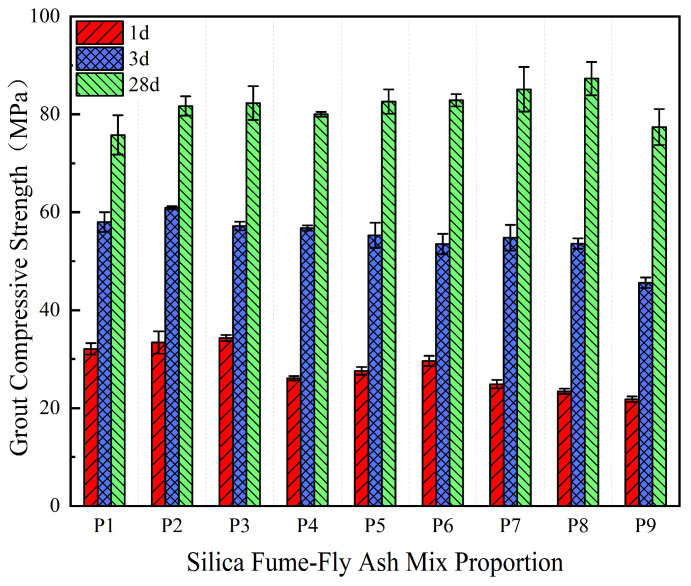
Effect of mineral admixture content on compressive strength.

Based on the orthogonal experimental results, the appropriate substitution ranges of cement by FA and SF were further investigated to evaluate their individual and combined effects on the workability and mechanical properties of the grouting material. The mix proportions for this investigation are listed in Table 13, and the corresponding test results for slump flow and compressive strength are presented in Figure 4 and Figure 5, respectively.

As shown in Figure 4, the slump flow decreases progressively with increasing silica fume content. This is primarily attributed to the fine particle size and large specific surface area of silica fume [34]. A higher SF content increases the total surface area of the cementitious materials, thereby raising the water demand of the mixture [35]. More water is thus consumed to wet the SF particle surfaces, leaving less free water available to maintain the fluidity of the fresh paste [36]. When the SF content is fixed, the slump flow increases with higher fly ash content. This improvement is mainly due to the spherical shape and smooth surface of fly ash particles, which reduce inter-particle friction and produce a “ball-bearing” effect [37]. Moreover, as the FA content increases, the cement proportion decreases, leading to a reduced water requirement and a significant lowering of the paste viscosity, both of which contribute to a higher slump flow [38]. Additionally, at a fixed silica fume content, increasing the fly ash content was generally associated with a larger slump flow diameter. This response may be related to changes in particle packing, particle morphology, interparticle friction, free-water availability, and superplasticizer demand.

It is evident from Figure 5 that the incorporation of an appropriate amount of silica fume is beneficial to the 1-day compressive strength. Compared with the mixture with 5.5% SF content (P1), the compressive strengths of grouts with 8.5% SF (P2) and 11.5% SF (P3) increased by 4% and 7%, respectively. However, for different fly ash contents, there exists an optimal silica fume dosage. Both SF and FA are mineral admixtures with pozzolanic activity, but the former possesses a larger specific surface area and contains substantial amorphous SiO_2_, which enables it to hydrate jointly with cement at the early stage [39]. Consequently, the early-age compressive strength of the grout is significantly enhanced. Nevertheless, when the silica fume content is excessive, insufficient water for early-age hydration will prevent the SF from fully reacting, which explains why the 3-day compressive strength at 11.5% SF (P3) decreased by 6% compared with that at 8.5% SF (P2).

A comparison among P1, P4, and P7 reveals that the incorporation of fly ash is detrimental to the 1-day compressive strength. Relative to the mixture with 12% FA (P1), the 1-day compressive strengths of grouts with 15% FA (P4) and 18% FA (P7) decreased by 19% and 22%, respectively. This is attributed to the pozzolanic effect of fly ash particles, which renders them less reactive during the early hydration of cement [40]. When the silica fume content is fixed at 5.5%, increasing the fly ash content promotes the 28-day compressive strength. Compared with P1, the 28-day strengths of P4 and P7 increased by 9% and 12%, respectively. The underlying mechanism is that fly ash replaces a portion of cement, reducing the early heat-flow rate and cumulative heat release during the first 72 h [41]. As cement hydration continuously generates sufficient Ca(OH)_2_, it provides an adequate reaction medium for fly ash containing active SiO_2_ and Al_2_O_3_, enabling the pozzolanic reaction to proceed thoroughly and generate substantial dense C-S-H gel [42]. Moreover, continued pozzolanic reaction at later ages may generate additional hydration products and contribute to the observed later-age strength development. However, excessive replacement of cement by both SF and FA, as in mix P9, also leads to a reduction in compressive strength, again demonstrating that there exists an optimal substitution level for both silica fume and fly ash.

**Figure 6:**
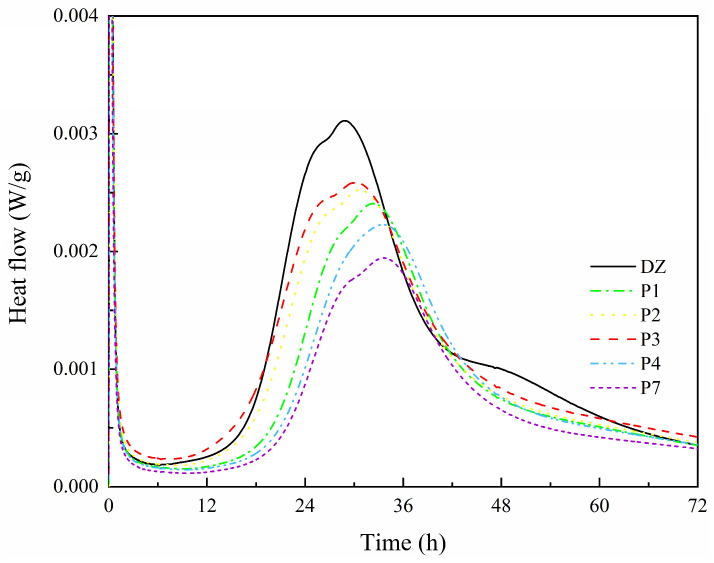
Effect of mineral admixture content on hydration heat release rate.

**Figure 7:**
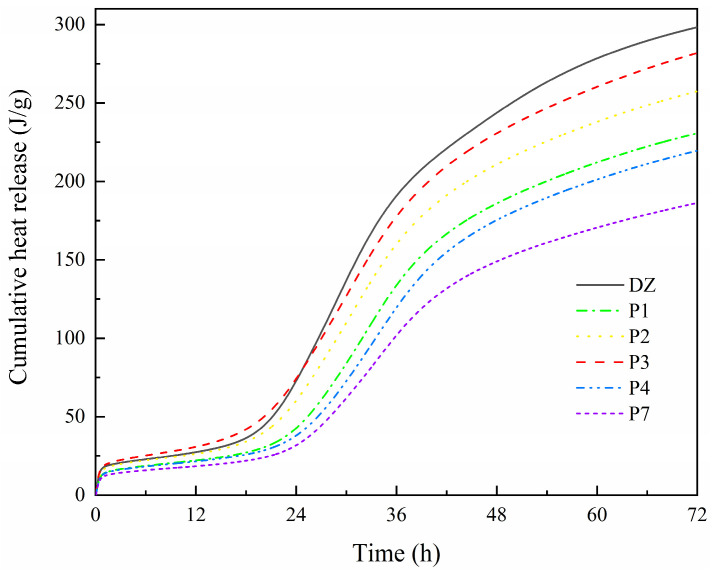
Effect of mineral admixture content on cumulative hydration heat.

Figure 6 and Figure 7 present the heat-flow and cumulative-heat curves of mixtures P1, P2, P3, P4, and P7, together with those of the cement-only reference-mixture DZ, during the first 72 h after water addition. In DZ, Portland cement accounted for 100% of the binder, without the incorporation of fly ash or silica fume. As shown in Figure 6, the main heat-flow peak of DZ occurred at approximately 28 h, with a peak value of approximately 3.1 mW/g, which was the highest among the tested mixtures. Because the binder in DZ consisted entirely of cement, no clinker-dilution effect associated with mineral-admixture replacement occurred. Consequently, its early hydration was more concentrated, resulting in an earlier and higher main heat-flow peak. The incorporation of fly ash significantly delays the induction period of cement hydration, and this delaying effect becomes more pronounced with increasing fly ash content [43]. The peak heat-release rate of mixture P7 appeared 12 h later than that of P3. This is because increasing the fly ash content was associated with a delayed and reduced main heat-flow peak. This trend may mainly reflect the dilution of Portland cement and the relatively low early-age reactivity of fly ash [44].

As observed in Figure 7, the incorporation of silica fume increases the total hydration heat of the cementitious materials. Compared with P1 (5.5% SF), the cumulative hydration heat of P2 and P3 increased by 9% and 32%, respectively. This result satisfactorily explains why P3, with the same FA content gradient, exhibits significantly higher 1-day compressive strength than mixtures with lower SF content. Under the incorporation of 18% fly ash (P7), the hydration heat before 40 h was the lowest among all mixtures, but showed an increasing trend at later stages. This growth rate is mainly governed by the FA content, which explains why P7 exhibited the lowest 1-day compressive strength. Furthermore, the hydration heat test results indicate that there exists an optimal range for the combined content of SF and FA in the cementitious system. Considering the requirements for fluidity, early-age compressive strength, and long-term compressive strength, the mineral-admixture proportioning tests yielded the more appropriate contents of 12% for fly ash and 8.5% for silica fume.

**Figure 8:**
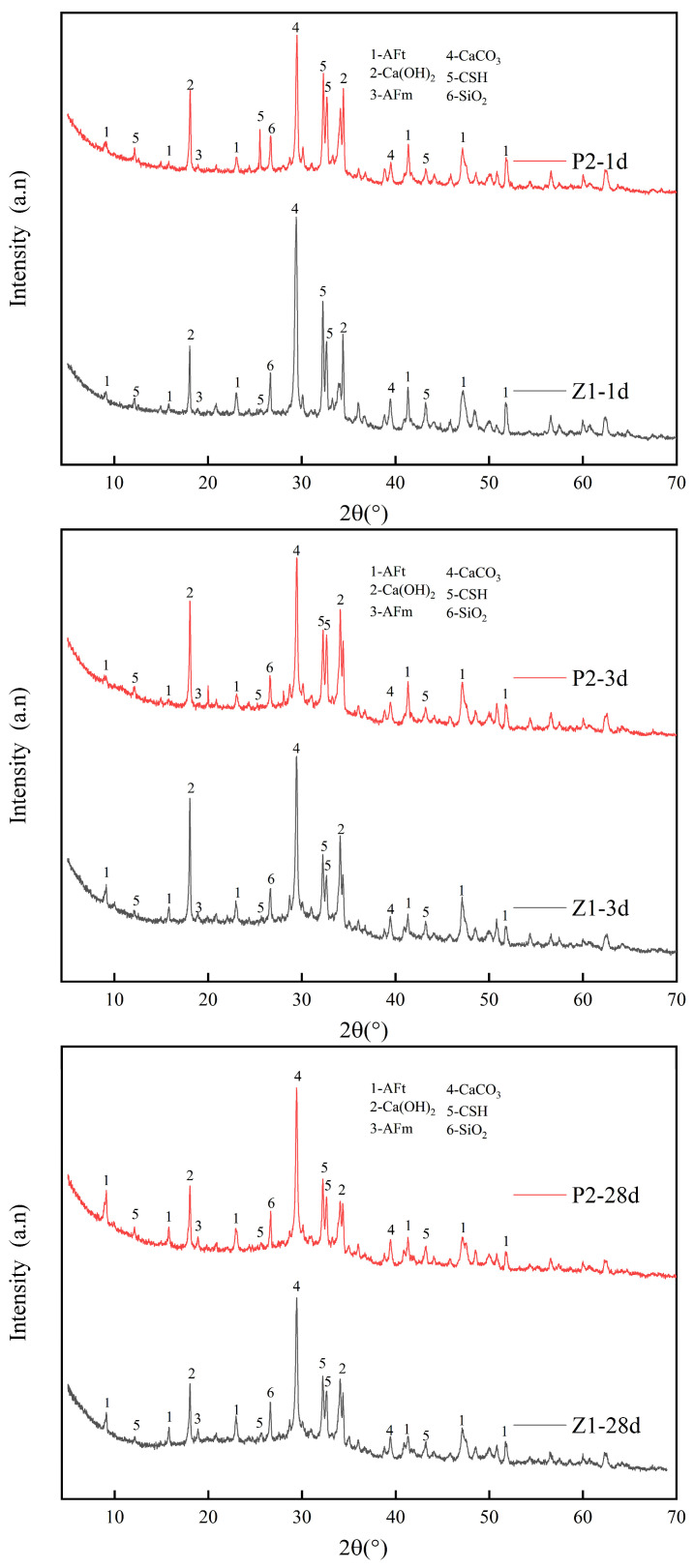
XRD patterns of Z1 and P2 at 1, 3, and 28 days.

**Figure 9:**
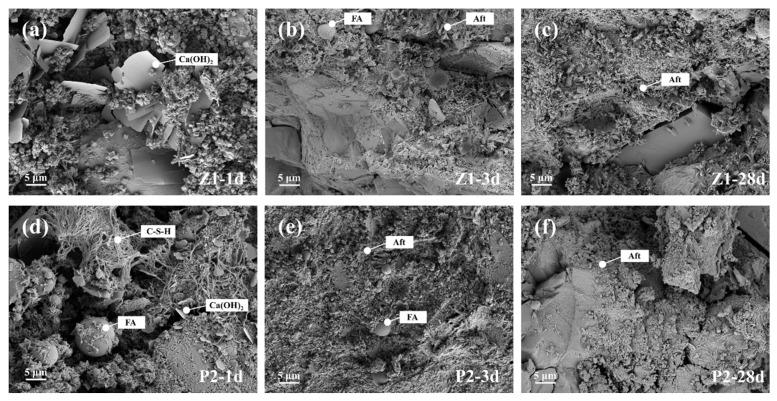
Comparative SEM images of mixes: (**a**) Z1-1d, (**b**) Z1-3d, (**c**) Z1-28d, (**d**) P2-1d, (**e**) P2-3d, (**f**) P2-28d.

From the SEM images and XRD analysis in Figure 8 and Figure 9, showing the results of the optimized best-factor-level-combination Z1 at different curing ages compared with those of the P2 mix, it can be observed that abundant plate-like crystals are present, which may be associated with portlandite based on their morphology. The qualitative XRD results, shown in Figure 9a, confirm that Z1 tends to enrich at the interface between the cement paste and aggregate and crystallize into coarse particles, thereby leading to a deterioration in interfacial bonding performance [45]. This interface consequently becomes a weak zone in the cement-based material, making it difficult to support the improvement of material strength. In contrast, for the optimized P2 mix shown in Figure 9d, the CH-related diffraction features appear less pronounced in the selected pattern, which accelerated the hydration reaction of the grout at an early age, promoting the generation of substantial C-S-H gel and needle-like ettringite and forming a more continuous network of hydration products in the selected field. The selected mixture showed less pronounced plate-like features that may be associated with portlandite, together with more extensive gel-like hydration products in the observed field. This may reflect two distinct processes: a possible nucleation effect of silica-fume particles on primary cement hydration and the later pozzolanic consumption of portlandite to form additional secondary C-S-H. Portlandite consumption itself should not be interpreted as direct evidence of accelerated clinker hydration. This ultimately resulted in a more significant improvement in the 1-day compressive strength of P2 compared with that of Z1.

As a moderately reactive admixture, fly ash exhibits an extremely low reaction degree at 1 day, basically retaining its original spherical particle morphology with only slight dissolution reactions occurring on the surface. As can be observed from Figure 8 and Figure 9e, the reaction of fly ash had also initiated, but its reaction rate was significantly slower than that of silica fume, with only a thin layer of C-S-H gel forming on the particle surface while the interior remained structurally intact. In Figure 9e, a thick layer of C-S-H gel had formed on the surface, and some silica fume particles had completely reacted, integrating into the overall hydration product network. At later ages, as shown in Figure 9f, the silica fume had essentially fully reacted. Its high pozzolanic activity enabled it to react thoroughly with Ca(OH)_2_, generating substantial secondary C-S-H gel and significantly enhancing the density of the microstructure [46]. The reaction degree of fly ash was also markedly improved, with most particles in Figure 9c being enveloped by thick layers of C-S-H gel and the particle edges becoming blurred, indicating that deep pozzolanic reactions had occurred in the interior.

## 4. Conclusions

This study systematically investigated the effects of water-to-binder ratio, fly ash content, total binder content, and silica fume content on the performance of Type IV cement-based grouting materials through orthogonal experiments and mineral admixture compounding tests. The initial and 30 min slump flows, as well as the 1-day, 3-day, and 28-day compressive strengths, were adopted as evaluation indicators. The following conclusions can be drawn:

(1) The silica fume content exerted the greatest influence on the initial slump flow. The water-to-binder ratio was the dominant factor affecting the 30 min slump flow and 1-day compressive strength. For the 3-day compressive strength, the water-to-binder ratio and silica fume content were the two principal factors. At 28 days, the corrected range and ANOVA results consistently indicated the order C > D > B > A, showing that the total binder content was the dominant factor, followed by the silica fume and fly ash contents, while the water-to-binder ratio had the smallest effect within the investigated range.
(2) Based on the multi-response evaluation of initial and 30 min slump flow and compressive strength at 1, 3, and 28 days, A_3_B_2_C_4_D_2_ was selected as the recommended compromise combination for the orthogonal stage. The corresponding verification Z1 mixture achieved initial and 30 min slump flows of 855 and 825 mm, respectively, and 1-day, 3-day, and 28-day compressive strengths of 23.7, 54.8, and 80.2 MPa, respectively. These results confirm that the recommended combination provides a satisfactory balance between flow retention, early-age strength, and later-age strength.
(3) In the supplementary tests conducted at a fixed water-to-binder ratio of 0.30 and a total binder content of 630 kg/m^3^, increasing silica fume content generally reduced slump flow but improved strength at selected curing ages. Increasing fly ash content improved flowability, reduced 1-day strength, and enhanced 28-day strength in some mixtures. Among the tested combinations, 12% fly ash and 8.5% silica fume provided the most favorable overall balance between workability and strength development.
(4) Compared with the cement-only reference used in the calorimetry tests, the fly ash–silica fume mixtures exhibited lower heat release during the first 72 h. Increasing silica fume content accelerated the early hydration process and increased heat release, whereas increasing fly ash content delayed the main hydration peak and reduced the early heat-release rate. The XRD and SEM observations indicated more extensive C-S-H formation and matrix densification at later ages in the selected blended mixtures. These observations support the different and complementary roles of silica fume and fly ash, but they should not be interpreted as quantitative evidence of statistical interaction effects.

## Figures and Tables

**Table 1:** Physical and mechanical properties of cement [5].

Loss on Ignition (%)	Setting Time (min)		Soundness	Flexural Strength (MPa)		Compressive Strength (MPa)	
	Initial	Final		3 d	28 d	3 d	28 d
4.47	231	286	Qualified	5.7	8.1	30.4	55.2

**Table 2:** Selected compositional indices of the cement.

Item	Clinker and Gypsum (%)	Loss on Ignition (%)	MgO (%)	SO_3_ (%)	Chloride (%)
Standard limit	80- ≤ 94	≤5.0	≤5.0	≤3.5	≤0.06
Measured value	89.5	3.52	2.89	3.13	0.016

**Table 3:** Physical properties of fly ash [5].

Fineness (0.045 mm Sieve Residue, %)	Water Demand (%)	Loss on Ignition (%)	SO_3_ (%)	Moisture Content (%)	Apparent Density (kg/m^3^)
6.7	93	2.5	0.8	0.3	2300

**Table 4:** Physical and chemical properties of silica fume [5].

Fineness (0.045 mm Sieve Residue, %)	SiO_2_ Content (%)	Water Demand (%)	Loss on Ignition (%)	Chloride Ion Content (%)	Moisture Content (%)	Activity Index (%)
2.1	92.04	104	1.24	0.008	0.8	108

**Table 5:** Factors and levels of the orthogonal experimental design.

Level	Water-to-Binder Ratio (A)	Fly Ash Content (B)/%	Total Binder Content (C)/kg/m^3^	Silica Fume Content (D)/%
1	0.27	9	540	2.5
2	0.29	15	590	8.5
3	0.31	21	640	14.5
4	0.33	27	690	20.5

**Table 6:** Compressive strength requirements for Type IV cementitious grout [20].

Age	A50/MPa	A60/MPa	A70/MPa	A85/MPa
1 d	≥15	≥20	≥25	≥35
3 d	≥30	≥40	≥45	≥60
28 d	≥50	≥60	≥70	≥85

**Table 7:** Mix design of orthogonal experimental test.

ID	Cement /kg/m^3^	Silica Fume /kg/m^3^	Fly Ash /kg/m^3^	10–20 mm/kg/m^3^	20–40 mm /kg/m^3^	70–120 mm /kg/m^3^	Water /kg/m^3^	5–16 mm Coarse Aggregate /kg/m^3^
1	477.9	13.5	48.6	503.8	274.8	137.4	145.8	801.3
2	451.4	50.2	88.5	485.1	264.6	132.3	159.3	771.7
3	412.8	92.8	134.4	466.5	254.5	127.2	172.8	742.0
4	362.3	141.5	186.3	447.9	244.3	122.1	186.3	712.4
5	451.4	85.6	53.1	481.7	262.7	131.4	171.1	766.2
6	348.3	110.7	81.0	500.6	273.0	136.5	156.6	796.2
7	527.9	17.3	144.9	443.8	242.1	121.0	200.1	706.0
8	412.8	54.4	172.8	462.7	252.4	126.2	185.6	736.1
9	451.2	131.2	57.6	459.0	250.4	125.2	198.4	730.1
10	486.5	100.1	103.5	439.8	239.9	119.9	213.9	699.5
11	380.7	45.9	113.4	497.4	271.3	135.7	167.4	791.2
12	416.0	14.8	159.3	478.2	260.8	130.4	182.9	760.6
13	569.3	58.7	62.1	435.7	237.7	118.8	227.7	693.1
14	528.0	16.0	96.0	455.2	248.3	124.2	211.2	724.1
15	345.2	121.0	123.9	474.7	258.9	129.5	194.7	755.1
16	315.9	78.3	145.8	494.2	269.6	134.8	178.2	786.2

**Table 8:** Orthogonal experimental design (L_16_(4^4^)) and test results.

No.	A	B	C	D	Test Results				
					Initial Slump Flow/mm	30 Min Slump Flow/mm	1 d Strength /MPa	3 d Strength /MPa	28 d Strength /MPa
1	0.27 (1)	9% (1)	540 (1)	2.5% (1)	430.0	265.0	46.8	64.5	80.0
2	0.27 (1)	15% (2)	590 (2)	8.5% (2)	495.0	345.0	40.0	52.9	76.6
3	0.27 (1)	21% (3)	640 (3)	14.5% (3)	300.0	200.0	29.8	45.3	72.6
4	0.27 (1)	27% (4)	690 (4)	20.5% (4)	300.0	200.0	22.8	36.4	76.8
5	0.29 (2)	9% (1)	590 (2)	14.5% (3)	480.0	320.0	33.9	46.0	76.2
6	0.29 (2)	15% (2)	540 (1)	20.5% (4)	342.5	245.0	25.4	38.1	61.1
7	0.29 (2)	21% (3)	690 (4)	2.5% (1)	860.0	817.5	25.9	41.5	79.0
8	0.29 (2)	27% (4)	640 (3)	8.5% (2)	900.0	690.0	26.3	43.3	69.9
9	0.31 (3)	9% (1)	640 (3)	20.5% (4)	370.0	230.0	24.5	37.7	69.7
10	0.31 (3)	15% (2)	690 (4)	14.5% (3)	670.0	560.0	27.4	41.6	79.2
11	0.31 (3)	21% (3)	540 (1)	8.5% (2)	347.5	340.0	27.3	45.6	79.7
12	0.31 (3)	27% (4)	590 (2)	2.5% (1)	835.0	792.5	17.2	42.7	68.0
13	0.33 (4)	9% (1)	690 (4)	8.5% (2)	895.0	860.0	23.4	47.3	79.5
14	0.33 (4)	15% (2)	640 (3)	2.5% (1)	870.0	850.0	21.5	40.5	62.4
15	0.33 (4)	21% (3)	590 (2)	20.5% (4)	405.0	365.0	14.9	30.5	69.2
16	0.33 (4)	27% (4)	540 (1)	14.5% (3)	735.0	627.5	15.6	30.4	74.5

**Table 9:** Range analysis of the slump flow results.

	Initial Slump Flow/mm					30 Min Slump Flow/mm				
	k1	k2	k3	k4	R	k1	k2	k3	k4	R
A	381.25	645.63	555.63	726.25	345.00	252.50	518.13	480.63	675.63	423.13
B	543.75	594.38	478.13	692.50	214.38	418.75	500.00	430.63	577.50	158.75
C	463.75	553.75	610.00	681.25	217.50	369.38	455.63	492.50	609.38	240.00
D	748.75	659.38	546.25	354.38	394.38	681.25	558.75	426.88	260.00	421.25
	Order of Significance: D > A > C > B					Order of Significance: A > D > C > B				

**Table 10:** Range analysis of the compressive strength results.

	1-Day Compressive Strength/MPa					3-Day Compressive Strength/MPa					28-Day Compressive Strength				
	k1	k2	k3	k4	R	k1	k2	k3	k4	R	k1	k2	k3	k4	R
A	34.85	27.87	24.10	18.85	16.00	49.77	42.22	41.90	37.17	12.60	76.5	71.55	74.15	71.40	5.10
B	32.15	28.58	24.48	20.47	11.68	48.88	43.27	40.73	38.20	10.68	76.35	69.83	75.13	72.30	6.53
C	28.77	26.50	25.52	24.88	3.90	44.65	43.03	41.70	41.70	2.95	73.83	72.50	68.65	78.63	9.98
D	27.85	29.25	26.67	21.90	7.35	47.30	47.27	40.83	35.67	11.63	72.35	76.43	75.63	69.20	7.23
	Order of Significance: A > B > D > C					Order of Significance: A > D > B > C					Order of Significance: C > D > B > A				

**Table 11:** Analysis of variance for slump flow and compressive strength.

Response	Factor	Sum of Squares	df	Mean Square	F-Value	*p*-Value
Initial slump flow	Water-to-binder ratio	519,772.656	3	173,257.552	51.703	*p* < 0.001
	Fly ash content	196,000.781	3	65,333.594	19.497	*p* < 0.001
	Total binder content	202,008.594	3	67,336.198	20.094	*p* < 0.001
	Silica fume content	697,972.656	3	232,657.552	69.429	*p* < 0.001
	Error	63,669.531	19	3351.028	—	—
30 min slump flow	Water-to-binder ratio	734,383.594	3	244,794.531	82.518	*p* < 0.001
	Fly ash content	109,039.844	3	36,346.615	12.252	*p* < 0.001
	Total binder content	244,633.594	3	81,544.531	27.488	*p* < 0.001
	Silica fume content	706,527.344	3	235,509.115	79.388	*p* < 0.001
	Error	56,364.844	19	2966.571	—	—
1 d compressive strength	Water-to-binder ratio	1635.495	3	545.165	85.471	*p* < 0.001
	Fly ash content	922.048	3	307.349	48.186	*p* < 0.001
	Total binder content	104.617	3	34.872	5.467	*p* = 0.003
	Silica fume content	369.445	3	123.148	19.307	*p* < 0.001
	Error	223.242	35	6.378	—	—
3 d compressive strength	Water-to-binder ratio	977.762	3	325.921	54.934	*p* < 0.001
	Fly ash content	746.752	3	248.917	41.955	*p* < 0.001
	Total binder content	70.627	3	23.542	3.968	*p* = 0.016
	Silica fume content	1138.355	3	379.452	63.956	*p* < 0.001
	Error	207.655	35	5.933	—	—
28 d compressive strength	Water-to-binder ratio	208.618	3	69.539	5.240	*p* = 0.004
	Fly ash content	308.025	3	102.675	7.737	*p* < 0.001
	Total binder content	612.635	3	204.212	15.388	*p* < 0.001
	Silica fume content	393.945	3	131.315	9.895	*p* < 0.001
	Error	464.473	35	13.271	—	—

**Table 12:** Residual error statistics for the investigated performance indicators.

Response	Error df	Error Mean Square	Residual Standard Deviation	Overall Mean	Residual Coefficient of Variation (%)	Residual Sum-of-Squares Proportion (%)
T_0_/mm	19	3351.028	57.89	577.19	10.03	3.8
T_30_/mm	19	2966.571	54.47	487.34	11.18	3.0
f_1 d_/MPa	35	6.378	2.53	26.42	9.56	6.9
f_3 d_/MPa	35	5.933	2.44	42.77	5.70	6.6
f_28 d_/MPa	35	13.271	3.64	73.40	4.96	23.4

**Table 13:** Mix proportions with different mineral admixture contents.

Mix No.	Silica Fume Content (%)	Fly Ash Content (%)
P1	5.50%	12.00%
P2	8.50%	12.00%
P3	11.50%	12.00%
P4	5.50%	15.00%
P5	8.50%	15.00%
P6	11.50%	15.00%
P7	5.50%	18.00%
P8	8.50%	18.00%
P9	11.50%	18.00%

## Data Availability

The original contributions presented in this study are included in the article. Further inquiries can be directed to the corresponding author.
